# Hypoxia Enhances HIF1*α* Transcription Activity by Upregulating KDM4A and Mediating H3K9me3, Thus Inducing Ferroptosis Resistance in Cervical Cancer Cells

**DOI:** 10.1155/2022/1608806

**Published:** 2022-03-05

**Authors:** Jing Xiong, Meifang Nie, Chun Fu, Xiaoshan Chai, Yongjing Zhang, Ling He, Shujuan Sun

**Affiliations:** Department of Obstetrics and Gynecology, The Second Xiangya Hospital of Central South University, Changsha, Hunan 410011, China

## Abstract

**Objective:**

Cervical cancer (CC) is a prevalent cancer in women. Hypoxia plays a critical role in CC cell ferroptosis resistance. This study explored the mechanism of hypoxia in CC cell ferroptosis resistance by regulating HIF1*α*/KDM4A/H3K9me3.

**Methods:**

Cultured SiHa and Hela cells were exposed to CoCl2 and treated with Erastin. Cell viability was detected by MTT assay, and concentrations of iron ion, MDA and GSH were determined using corresponding kits. Expressions of KDM4A, HIF1*α*, TfR1, DMT1, and H3k9me3 were detected by RT-qPCR, Western blot, and ChIP assay. The correlation of KDM4A and HIF1*α* was analyzed on Oncomine, UALCAN, and Starbase. CC cells were co-transfected with shKDM4A or/and pcDNA3.1-HIF1*α*. Iron uptake and release were assessed using the isotopic tracer method. The binding relationship between HIF1*α* and HRE sequence was verified by dual-luciferase assay.

**Results:**

Cell viability and GSH were decreased while iron concentration, MDA, KDM4A, and HIF1*α* levels were increased in hypoxia conditions. The 2-h hypoxia induced ferroptosis resistance. KDM4A and HIF1*α* were highly-expressed in CC tissues and positively correlated with each other. KDM4A knockdown attenuated cell resistance to Erastin, increased H3K9me3 level in the HIF1*α* promoter region, and downregulated HIF1*α* transcription and translation. H3K9me3 level was increased in the HIF1*α* promoter after hypoxia. HIF1*α* overexpression abrogated the function of KDM4A knockdown on ferroptosis in hypoxia conditions. Iron uptake/release and TfR1/DMT1 levels were increased after hypoxia. Hypoxia activated HRE sequence in TfR1 and DMT1 promoters.

**Conclusion:**

Hypoxia upregulated KDM4A, enhanced HIF1*α* transcription, and activated HRE sequence in TfR1 and DMT1 promoters via H3K9me3, thus inducing ferroptosis resistance in CC cells.

## 1. Introduction

As one of the most common types of malignancies in women, cervical cancer (CC) is linked to high incidence and mortality second only to breast cancer [1, 2]. China sees particularly high mortality and incidence, with about 75000 females diagnosed with CC each year, of whom 40000 die from it [3]. Females at lower ages are more susceptible to CC [4]. The public regards CC as one of the dominant public health problems and a substantial burden of disease especially in developing and underdeveloped countries due to restrained medical conditions [5]. Therefore, it is of paramount importance to develop novel therapeutic strategies for CC.

Ferroptosis is a new type of iron-dependent programmed cell death that varies from apoptosis, necrosis, and autophagy [6]. Increased free iron, lipid peroxidation, and glutathione depletion are distinguishable characteristics of ferroptosis [7, 8]. It's important to note that ferroptosis has unique functions in cancer immunotherapy [9]. For example, ATF2 increases CC cell survival by attenuating ferroptosis [10]. Ferroptosis inducers, including small molecule compounds (such as Erastin) or drugs (such as sulfasalazine, sorafenib, and artesunate) produce inhibitory effects on tumor growth by provoking cell death [11, 12]. Conversely, tumor cell survival can be suppressed by hypoxia-induced factor 1*α* (HIF1*α*), a subunit of HIF-1 and a heterodimeric transcription factor, showing close association with the local suppression of anti-tumor immune responses [13–15]. It has been reported that HIF1*α* plays a key part in CC [16]. Hypoxia response element (HRE) is a significant regulatory sequence mediating hypoxic responses that interact with HIF1 in the nucleus [17]. Transferrin receptor 1 (TfR1) and divalent metal transporter 1 (DMT1) are both target genes of HIF1 [18]. Therefore, it's reasonable to assume that HIF1*α* might function in CC with the engagement of HRE, TfR1, and DMT1.

Lysine (K)-specific demethylase 4A (KDM4A/JMJD2A) is a member of the Jumonji domain 2 histone demethylases family [19] that catalyses histone demethylation from lysine residues, transcriptionally regulates gene expressions in tumor cells, and serves as a potential therapeutic target for cancers [20–22]. For example, Yan et al. have reported that JMJD2A was upregulated in human CC cells and cervical epithelial cancer tissues, which further impeded cancer cell apoptosis, and JMJD2A upregulation was closely correlated with poor overall and disease-free survival rate [23]. Nonetheless, the present understanding of KDM4A in CC as a carcinogenic protein is limited. Hypoxic areas are common birthplaces of solid tumors [24]. However, the functional mechanism of KDM4A in CC cell ferroptosis under hypoxic conditions has not been described. Histone H3 lysine 9 trimethylation (H3K9me3) is a pivotal epigenetic mechanism suppressing gene expressions [25]. Once KDM4A is depleted or deactivated, H3K9me3 accumulates at the HIF1*α* site, leading to HIF1*α* downregulation and stability decline [26]. Loss of SUV39H1 manipulates the H3K9me3 status of the DPP4 gene promoter to raise its expression, thus contributing to ferroptosis [27]. Thereby, this study outlines the functional mechanism by which KDM4A regulates CC cell ferroptosis under hypoxia by mediating HIF1*α* and HRE in TfR1 and DMT1 promoters through H3K9me3.

## 2. Materials and Methods

### 2.1. Bioinformatics Analysis

The expression of KDM4A in the CC dataset (http://www.ncbi.nlm.nih.gov/geo/query/acc.cgi?acc=GSE7803) was inquired on the Oncomine database (https://www.oncomine.org/resource/login.html). CC data in the cancer genome atlas (TCGA) database were analyzed using the UALCAN website (http://ualcan.path.uab.edu/index.html) with HIF1*α* expression profile analysis obtained. The correlation between KDM4A mRNA expression and HIF1*α* mRNA expression in CC tissues was analyzed using the ENCORI (http://starbase.sysu.edu.cn/panGeneCoExp.php).

### 2.2. Cell Culture

Hela cell line was provided by the Institute of Basic Medical Sciences Chinese Academy of Medical Sciences (Beijing, China) and cultured in DMEM (Gibco, Grand Island, NY, USA) containing 10% fetal bovine serum (FBS). SiHa cell line was procured from Shanghai Institutes for Biological Sciences (Shanghai, China) and maintained in DMEM high-glucose complete medium (Gibco) containing 10% FBS. The cells were cultured in an incubator at constant temperature of 37°C with 5% CO_2_ in the air. Upon 80% confluence, cells were detached with 0.025% trypsin (Gibco) and passaged.

### 2.3. Cell Treatment

The hypoxia and normoxia treatment procedures were as follows. Firstly, 100 *μ*L SiHa and Hela cells were seeded in 96-well plates (5 × 10^4^ cells/mL). According to the different culture conditions, the cells were allocated to the normoxia group (Normal) (cells cultured at the constant temperature at 37°C with 5% CO_2_) and hypoxia group (Hypoxia) (cells cultured in the medium containing 100 *μ*mol/L CoCl_2_ in an incubator with constant temperature at 37°C with 5% CO_2_ for 0.5, 1, 2, 4 and 12 h). After 2-h culture, cells were added with Erastin (HY-15763; MCE, Monmouth Junction, NJ, USA) at the final concentration of 5 *μ*mol/L and incubated for 24 h [28].

### 2.4. Cell Transfection

SiHa and Hela cells (5 × 10^4^) were seeded in 12-well plates and cultured until reaching 70-80% confluence. The shRNAs of KDM4A (shKDM4A-1, shKDM4A-2, shKDM4A-3), pcDNA3.1-HIF1*α*, and corresponding controls were delivered into cells using Lipofectamine 2000 (Invitrogen, Carlsbad, CA, USA) for 24 h, followed by 2-h culture in normoxic or hypoxic conditions and 24-h treatment with Erastin. Cells were grouped as follows: the Normal + Erastin + shNC group, the Normal + Erastin + shKDM4A group, the Hypo-2 h + Erastin + shNC group, the Hypo-2 h + Erastin + shKDM4A group, the Hypo-2 h + shKDM4A + pcDNA3.1-HIF1*α* group, and the Hypo-2 h + shKMD4A + pcDNA3.1 group. The overexpression and interference plasmids of KDM4A and their controls were provided by GenePharma (Shanghai, China) and pcDNA3.1-HIF1*α* and pcDNA3.1 were supplied by Hanbio Biotechology (Shanghai, China).

### 2.5. MTT Assay

Cell activity was detected using 3-(4,5-dimethylthiazol-2-yl)-2,5-diphenyl-2H-tetrazolium bromide (MTT) method. Cells in each well were added with 200 *μ*L serum-free medium and 20 *μ*L MTT reagent (5 mg/mL), cultured at 37°C with 5% CO_2_ for 4 h, and centrifuged at 1000 r/min for 5 min to discard the supernatant. Then, cells in each well were added with 150 *μ*L dimethyl sulfoxide (DMSO) and shaken for 10 min. The optical density at 490 nm was measured using a microplate reader. At the same time, the blank group (containing culture medium only) was set up for correction. Cell activity = experimental group - blank group/control group - blank group.

### 2.6. GSH and Iron Ion Concentration Determination

Glutathione (GSH) and iron ion concentration were determined using Glutathione Colorimetric detection kit (ARBOR ASSAYS, Michigan, USA) and Iron Colorimetric Assay kit (Applygen, Beijing, China) as instructed by the manufacturer's protocols.

### 2.7. MDA Determination

Proteins from cells or tissues were extracted using lysate, and the protein concentration was determined using the bicinchoninic acid (BCA) method. Thiobarbituric acid reaction was performed using lipid malondiadehycle (MDA) assay kits (Merck, KGaA, Darmstadt, Germany) to measure MDA concentration in cells or tissue lysates. The absorbance at 532 nm was measured using a microplate reader, and the MDA level was normalized to protein concentration.

### 2.8. Total RNA Extraction and RT-qPCR

The cervical tissues were ground in liquid nitrogen. The total RNA was extracted from tissues or cells using TRIzol reagent (Invitrogen). RNA concentration and purity were determined using ultraviolet absorption method. The total RNA was transcribed into cDNA using ReverTra Ace qPCR RT Kit (FSQ-301, TOYOBO, Shiga, Japan). Reverse transcription quantitative polymerase chain reaction (RT-qPCR) was performed using SYBR® Premix Ex TaqTM II (Takara, Dalian, China) on ViiA 7 real-time PCR system (Applied Biosystems, Foster City, CA, USA) under following conditions: pre-denaturation at 94°C for 1 min and 35 cycles of denaturation at 55°C for 1 min, annealing at 72°C for 1 min and extending at 72°C for 5 min, with *β*-actin as the internal reference. The data were analyzed using the 2^-*ΔΔ*CT^ method. Amplified primer sequences were shown in Table 1.

### 2.9. ChIP Assay

Chromatin immunoprecipitation (ChIP) assay was performed using Simple ChIP Enzymatic Chromatin IP kit (Cell Signaling Technology, USA) in line with the manufacturer's protocols. After cell crosslinking and lysis, the DNA was fragmented to 200-1000 bp in length on ice by ultrasound. The cells were centrifuged to collect the supernatant. The samples were added with H3K9me3 antibody (ab8898, 4 *μ*g for 25 *μ*g of chromatin, Abcam, Cambridge, MA, USA) for immunoprecipitation with rabbit IgG (ab46540, Abcam) as the negative control. The chromatin after ultrasonic fragmentation was used as the Input control. The antibody-protein complexes were harvested for a series of elution and decrosslinking. DNA fragments were purified and enriched using RNase A, EDTA, Tris and proteinase K. The relative expression of the HIF1*α* promoter region was detected by RT-qPCR using specific primers.

### 2.10. Western Blot

The tissues were ground in liquid nitrogen. The total protein was extracted from tissues or cells using radio-immunoprecipitation assay lysis buffer (Solarbio, Beijing, China). BCA Protein Assay kit (Beyotime, Shanghai, China) was utilized to quantify protein. Electrophoresis was performed in a cold room at the temperature of 4°C at 80 V for 90 min. Afterwards, the protein was transferred to polyvinylidene fluoride (PVDF) membranes using the wet electroporation method at 100 mA for 90 min. PVDF membranes were washed on a shaker for 5 min and sealed with 5% skim milk-tris buffered saline and Tween-20 (TBST) for 1 h. Subsequently, the samples were shaken in primary working fluid overnight at 4°C. The primary antibodies were listed as follows: rabbit HIF1*α* antibody (1 : 1000, 93KD, ab51608, Abcam), rabbit KDM4A antibody (1 : 5000, 150KD, ab191433, Abcam), rabbit H3K9me3 antibody (ab8898, 15KD, 1 : 1000, Abcam), rabbit TfR1 antibody (1 : 1000, 85KD, ab214039, Abcam), rabbit DMT1 antibody (2 *μ*g/mL, 62KD, ab55812, Abcam) and rabbit *β*-actin antibody (1 : 1000, 41KD, ab252556, Abcam). On the following day, the PVDF membranes were washed using TBST thrice, soaked in secondary antibody horseradish peroxidase-labeled goat anti-rabbit IgG (1 : 10000, ab6721, Abcam) working fluid, and incubated at room temperature for 1 h. After retrieving the secondary antibody working fluid, the samples were washed 3 times with TBST. The developer was spread on the PVDF membranes to develop for 1-3 min. With *β*-actin as the internal reference, the protein bands were visualized by chemiluminescence and gray value was analyzed.

### 2.11. Determination of Iron Uptake and Release by Isotope Tracer Method

Iron uptake was determined as described. After 3 washes with phosphate buffer saline (PBS), cells were incubated with 1 *μ*mol/L ^55^FeCl3 [1 mL sucrose incubation solution +16 *μ*L 62.5 *μ*mol/L ^55^FeCl3 (Perkinelmer, Waltham, MA, USA)] at 37°C for 30 min, and washed with cold PBS thrice. Then, the cells were added with 500 *μ*L 1% sodium dodecyl sulfate (SDS) solution for 10 min to lyse cells, and 300 *μ*L lysate was added into 2 mL scintillator. Finally, the counts per minute (cpm) were measured using a liquid scintillation counter (Perkinelmer, 1450), and the remaining 200 *μ*L lysate was used for protein quantification using the BCA method. The iron content per *μ*g protein was estimated.

Iron release was measured as mentioned. After 3 PBS washes, cells were incubated with 1 *μ*mol/L ^55^FeCl3 (1 mL sucrose incubation solution +16 *μ*L 62.5 *μ*mol/L ^55^FeCl3) at 37°C for 30 min, and washed with cold PBS 2 times. Then, the cells were added with 500 *μ*L 1% SDS solution for 10 min to lyse cells, and 300 *μ*L lysate was added into 2 mL scintillator. The cpm was measured using a liquid scintillation counter (Perkinelmer, 1450). The release of ^55^Fe (%) = supernatant cpm value/(supernatant cpm value + cell lysate cpm value) ×100%.

### 2.12. Dual-Luciferase Assay

Referring to the previous studies [29, 30], TfR1-HRE-Luc and DMT1-HRE-Luc luciferase reporter vectors were constructed by cloning of 5'-regulatory region fragment using PCR with human HepG2 cell genomic DNA as a template. The sequences of TfR1-HRE primers for PCR cloning were: forward 5'-CGGGGTACCAGGCTACCAGGGTGGAGGAA-3', reverse 5'-CCGCTCGAGACGCTGAGGGGATGGC-3'. The sequences of DMT1-HRE were: forward 5'-TGGCCTGGCTACCCTTTAC-3', reverse 5'-AGTTGCTGCTTGCGTTGG-3'. The luciferase reporter vectors TfR1-HRE-Luc and DMT1-HRE-Luc were constructed by inserting the fragment into pGL3-Basic (Promega, Madison, WI, USA). The coding sequence of HIF1*α* obtained by PCR was: forward 5'-ATGGAGGGCGCCGGCGGCGAG-3', reverse 5'-GTTAACTTGATCCAAAGCTCTGAG-3'. The pcDNA3.1-HIF1*α* plasmid was obtained by inserting the fragment into the pcDNA3.1 (+) plasmid. All the luciferase plasmids were transfected following the manufacturer's instructions of FuGENE HD (Roche Diagnostics, IN, USA). Once cell confluence reached 90%, the constructed plasmids were co-transfected with the internal reference vector PRL-TK (Promega) (100 : 1). Subsequently, cells were harvested and the luciferase activity was measured using a microplate reader (Bio-Rad 680, Bio-Rad, Hercules, CA, USA).

### 2.13. Statistical Analysis

SPSS21.0 statistical software (IBM Corp. Armonk, NY, USA) was employed for data analysis. The data were expressed as mean ± standard deviation. An independent *t* test was to compare data between 2 groups, and one-way analysis of variance (ANOVA) was adopted to compare data among multi-groups. Tukey's multiple comparison test was utilized as the post hoc test. The *p* value was obtained by a bilateral test, where *p* <0.05 indicated statistical significance.

## 3. Results

### 3.1. Hypoxia Inhibited CC Cell Viability and Induced Ferroptosis Resistance

**Figure 1:**
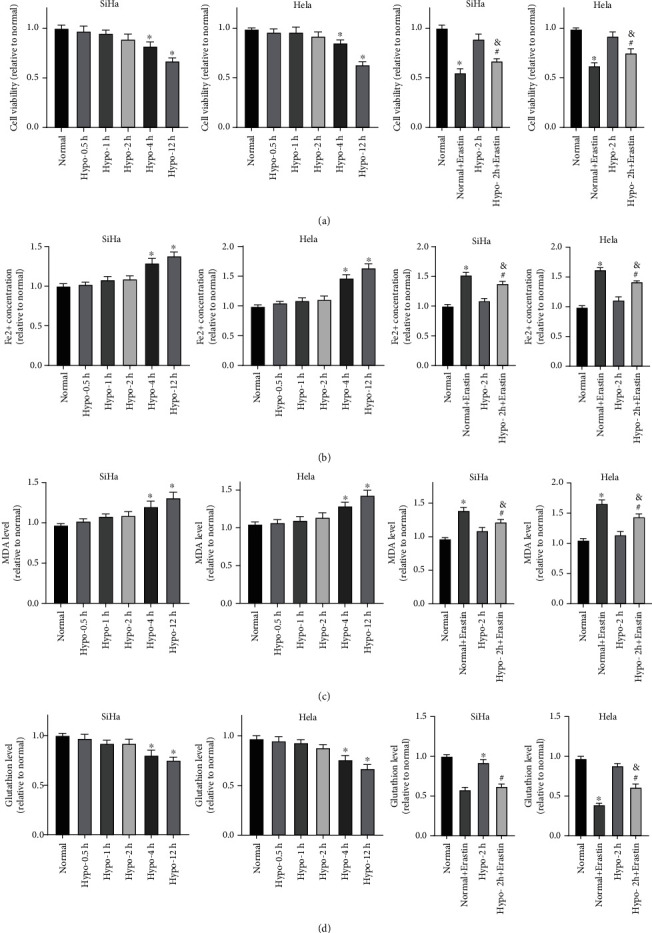
Hypoxia inhibited CC cell viability and induced ferroptosis resistance. SiHa and Hela cell lines were treated with hypoxia for 0.5, 1, 2, 4, and 12 h, or SiHa and Hela cell lines cultured in normoxic and hypoxic conditions for 2 h were co-incubated with ferroptosis inducer Erastin for 24 h. (A) Cell viability was determined by MTT assay; (B-D) The intracellular iron concentration, MDA, and GSH levels were measured using colorimetry method. The experiments were conducted in 3 replicates independently. The data were expressed as mean ± standard deviation. One-way ANOVA was adopted for comparisons among groups with Tukey's multiple comparison test as the post hoc test; ∗vs. the normal group, *p* <0.05; # vs. the Hypo-2 h group, *p* <0.05; & vs. the Normal + Erastin group, *p* <0.05.

Firstly, the effects of hypoxia on the viability of SiHa and Hela cells were explored. MTT assay demonstrated decreased viability of hypoxic cells compared with that of normoxic cells at all-time points. The survival rates of SiHa and Hela cells in hypoxic conditions for 0.5-2 h were not significantly different from those in the corresponding control groups, but were gradually decreased with the extension of hypoxia time (Figure 1(a), all *p* <0.05). To study the effect of hypoxia on ferroptosis of SiHa and Hela cells at different time points, the intracellular iron concentration, MDA level, and GSH level were determined. With the prolongation of hypoxia, iron ion concentration in SiHa and Hela cells was increased (Figure 1(b), all *p* <0.05), MDA level was increased (Figure 1(c), all *p* <0.05), and GSH level was decreased (Figure 1(d), all *p* <0.05). However, intracellular iron ion concentration, MDA, and GSH levels in cells treated with hypoxia for 0.5-2 h showed no evident difference from those in cells treated with normoxia. These results indicated that 2-h hypoxia treatment did not affect ferroptosis level. Thereby, the ferroptosis inducer Erastin was added in the normoxia group and 2 h-hypoxia treatment group. Compared with the Normal + Erastin group, the Hypo-2 h + Erastin group had increased cell viability (Figure 1(a), *p* < 0.05), decreased intracellular iron ion concentration and MDA level (Figure 1(b)–1(c), all *p* <0.05), and increased GSH level (Figure 1(d), *p*< 0.05). These results suggested that 2 h-hypoxia treatment induced ferroptosis resistance in SiHa and Hela cells.

### 3.2. Hypoxia Induced Upregulation of KDM4A in CC Cells

**Figure 2:**
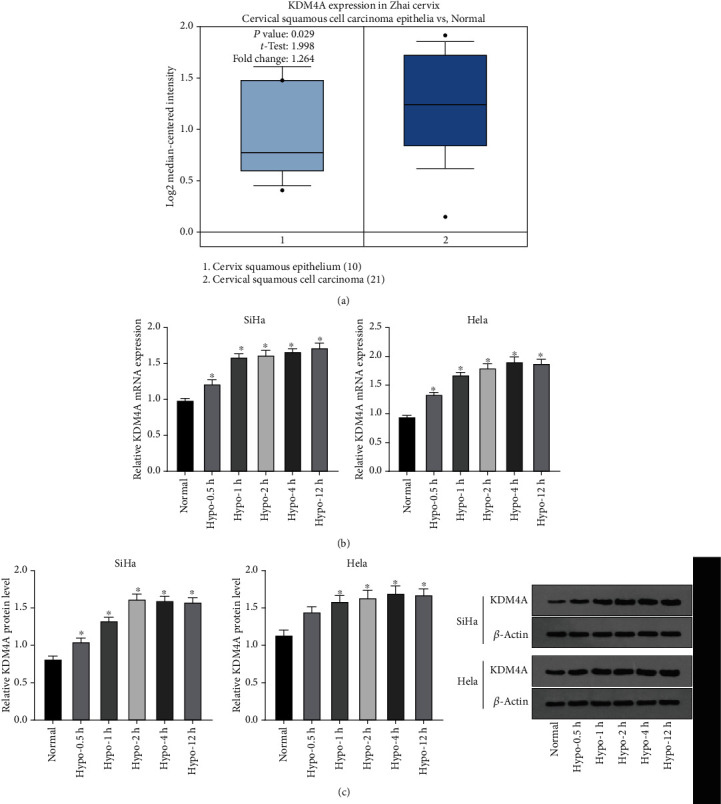
Hypoxia induced upregulation of KDM4A in CC cells. SiHa and Hela cell lines were treated with hypoxia for 0.5, 1, 2, 4, and 12 h. (A) The expression of KDM4A in CC research data set GSE7803 was obtained from the Oncomine database; (B-C) The mRNA and protein levels of KDM4A in CC cells were detected by RT-qPCR and Western blot. The experiments were conducted in 3 replicates independently. The data were expressed as mean ± standard deviation. One-way ANOVA was used for comparisons among groups with Tukey's multiple comparisons test as the post hoc test; ∗ vs. the normal group, *p* <0.05.

Researchers have reported that hypoxia regulates gene expressions through epigenetic mechanisms such as DNA methylation, non-coding RNA, and histone modification, thus promoting tumorigenesis and progression [31]. However, the epigenetic mechanism of ferroptosis is still elusive. We speculated that hypoxia-induced ferroptosis resistance might be in close relation to the epigenetic mechanism. KDM4A (JMJD2A) can catalyze the histone to remove methyl from lysine residues, and KDM4A can promote the growth of CC cells and inhibit their apoptosis [23]. By searching the Oncomine database, we found that KDM4A was highly expressed in cervical squamous cell carcinoma compared with that in the normal cervical epithelial tissues (Figure 2(a), *p*< 0.05). Subsequently, SiHa and Hela cells were treated with hypoxia for 0.5-12 h. RT-qPCR and Western blot showed that the mRNA and protein levels of KDM4A were upregulated with the extension of hypoxia treatment (Figure 2(b)-2(c), all *p* <0.05).

### 3.3. Hypoxia Mediated KDM4A Inducing Ferroptosis Resistance in CC Cells

**Figure 3:**
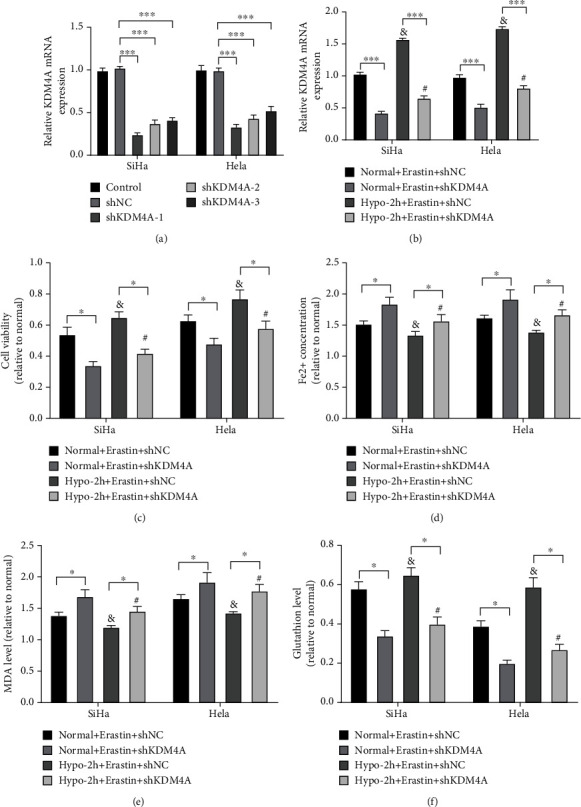
Hypoxia mediated KDM4A to induce ferroptosis resistance in CC cells. SiHa and Hela cell lines were transfected with shKDM4A, followed by 2-h normoxia or hypoxia treatment and 24-h incubation with Erastin. (A) The efficiency of shKDM4A transfection was detected by RT-qPCR; (B) The mRNA level of KDM4A was detected by RT-qPCR; (C) Cell viability was determined by MTT assay; (D-F) The intracellular iron concentration, MDA, and GSH levels were measured using colorimetry method. The experiments were conducted in 3 replicates independently. The data were expressed as mean ± standard deviation. One-way ANOVA was used for comparisons among groups with Tukey's multiple comparisons test as the post hoc test; ∗*p* < 0.05, & vs. the Normal + Erastin + shNC group, *p* <0.05; # vs. the Normal + Erastin + shKDM4A group, *p* <0.05.

We speculated that hypoxia treatment might induce ferroptosis resistance via regulating epigenetic gene KDM4A. Various shRNAs of KDM4A were used to transfect SiHa and Hela cell lines to identify the role of KDM4A in ferroptosis resistance, and the transfection efficiency was shown in Figure 3(a). shKDM4A-1 (named shKDM4A) with the highest interference efficiency was selected for subsequent experimentation. Following transfection, SiHa and Hela cell lines were treated with normoxia or hypoxia for 2 h and co-incubated with Erastin. KDM4A mRNA level was partially increased in hypoxia condition relative to normoxic condition, that is, in the Hypo-2 h + Erastin + shNC group relative to the Normal + Erastin + shNC group or in the Hypo-2 h + Erastin + shKDM4A group relative to the Normal + Erastin + shKDM4A group (Figure 3(b), all *p* <0.05). After KDM4A knockdown, the viability of SiHa and Hela cells was decreased (Figure 3(c), all *p* <0.05), Fe^2+^ and MDA were increased (Figure 3(d)-3(e), all *p* <0.05), and GSH level was decreased (Figure 3(f), all *p* <0.05), suggesting that KDM4A knockdown increased ferroptosis level of CC cells and inhibited ferroptosis resistance. In contrast to the Normal + Erastin + shNC group or the Normal + Erastin + shKDM4A group, the Hypo-2 h + Erastin + shNC group or the Hypo-2 h + Erastin + shKDM4A group had increased cell viability (Figure 3(c), all *p* <0.05), decreased Fe^2+^ and MDA (Figure 3(d)-3(e), all *p* <0.05), and increased GSH level (Figure 3(f), all *p* <0.05). These results further unraveled that hypoxia-mediated KDM4A induced ferroptosis resistance.

### 3.4. KDM4A Mediated H3K9me3 Enhancing the Transcriptional Activity of HIF1*α*

**Figure 4:**
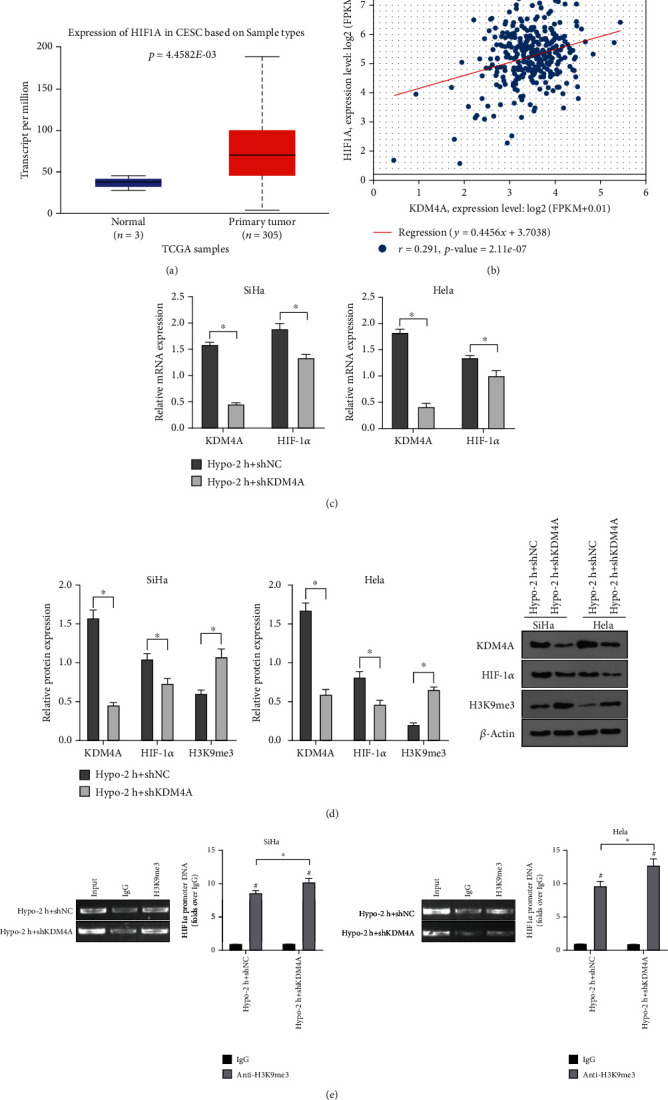
KDM4A mediated H3K9me3 to enhance the transcriptional activity of HIF1*α* in hypoxia. SiHa and Hela cell lines were interfered with using shKDM4A and treated with hypoxia for 2 h. (A) The analysis map of HIF1*α* expression profile in TCGA CC database was obtained from the UALCAN website; (B) ENCORI pan cancer coexpression analysis showed a positive correlation between KDM4A mRNA level and HIF1*α* mRNA level in CC cells; (C) The mRNA levels of KDM4A and HIF1*α* in CC cells were detected by RT-qPCR; (D) The protein levels of KDM4A, HIF1*α* and H3K9me3 in CC cells were detected by Western blot; (E) The level of H3K9me3 in HIF1A promoter was detected by ChIP assay. The experiments were conducted in 3 replicates independently. The data were expressed as mean ± standard deviation. The independent *t* test was used for comparison among groups. ∗*p* < 0.05, #compared with the IgG group, *P* <0.05. CESC: Cervical squamous cell carcinoma.

H3K9me3 is an important epigenetic mechanism inhibiting gene expression and is mediated by KDM4A to regulate in various tumor cells [32]. HIF1*α* is a composition of HIF-1, a key transcription regulator that mediates tumor cells to accommodate to hypoxic tumor microenvironment. We speculated the same regulatory mechanism in CC cells. By searching the CC dataset on the UALCAN website, we found that HIF1*α* was highly expressed in cervical squamous cell carcinoma tissues compared with that in normal cervical epithelial tissues (Figure 4(a)). Analysis of ENCORI pan cancer co-expression demonstrated a positive correlation between KDM4A mRNA and HIF1*α* mRNA level in CC tissues (Figure 4(b)). Subsequently, SiHa and Hela cell lines were transfected with shKDM4A and treated with hypoxia for 2 h. In the Hypo-2 h + shKDM4A group, RT-qPCR and Western blot showed that the mRNA and protein levels of KDM4A and HIF1*α* were decreased, while H3K9me3 level was increased (Figure 4(c)-4(d), all *p* <0.05), indicating that KDM4A knockdown decreased HIF1*α* level and increased H3K9me3 level in hypoxia. The chromatin was immunoprecipitated with immunoglobulin IgG or anti-H3K9me3 antibody. The purified DNA was amplified using specific primers of the HIF1*α* promoter region. H3K9me3 was enriched in the HIF1*α* promoter, and H3K9me3 level in the HIF1*α* promoter was elevated after KDM4A knockdown (Figure 4(e), all *p* <0.05). These results suggested that KDM4A was upregulated in CC cells in hypoxic conditions, and enhanced the transcriptional activity of HIF1*α* by downregulating H3K9me3.

### 3.5. Hypoxia Upregulated KDM4A/HIF1*α* Resistance to Ferroptosis in CC Cells

**Figure 5:**
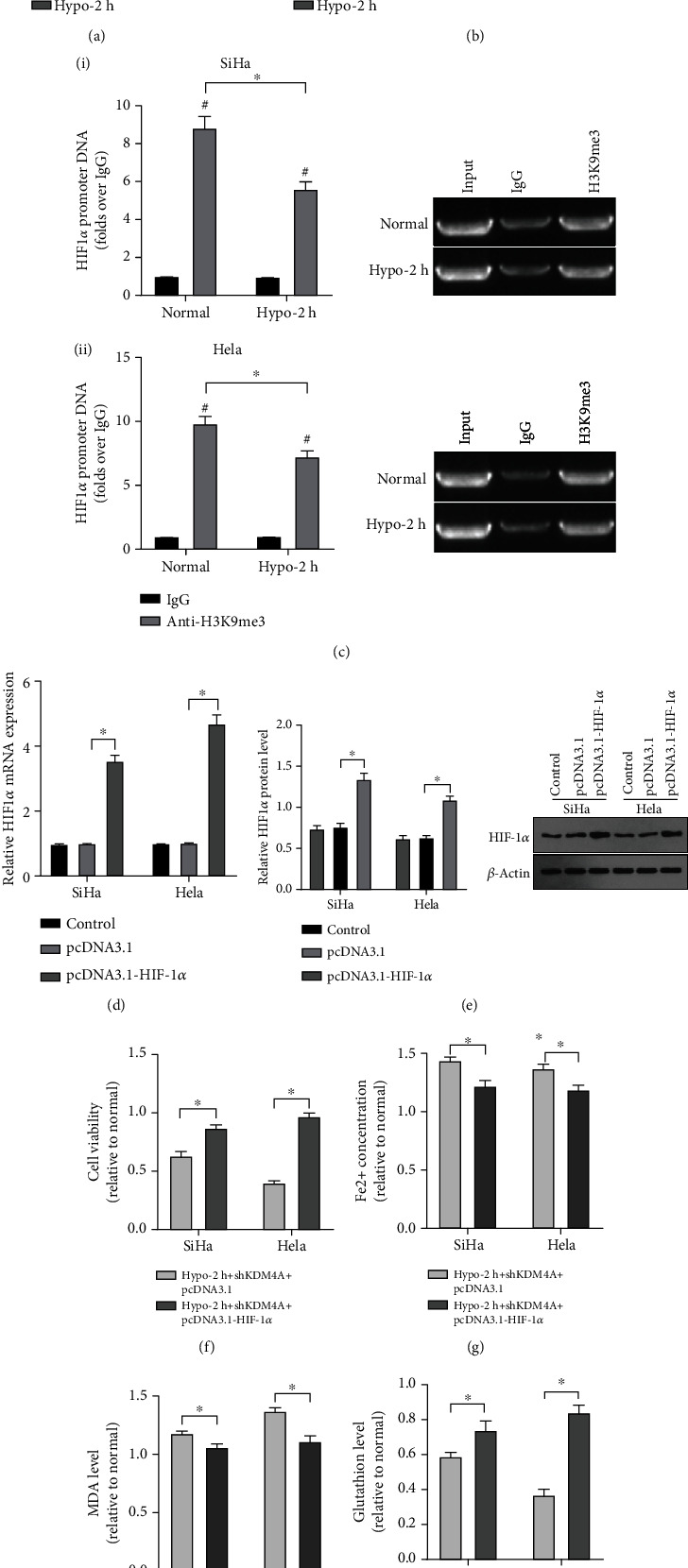
Hypoxia upregulated KDM4A/HIF1*α* resistance to ferroptosis in CC cells. SiHa and Hela cell lines were simultaneously transfected with shKDM4A and pcDNA3.1-HIF1*α*, followed by hypoxia treatment. (A-B) The mRNA and protein levels of HIF1*α* were detected by RT-qPCR and Western blot; (C) The level of H3K9me3 in HIF1A promoter was detected by ChIP assay; (D-E) The transfection efficiency of pcDNA3.1-HIF1*α* was detected by RT-qPCR and Western blot; (F) Cell viability was determined by MTT assay; (G-I) The intracellular iron concentration, MDA and GSH levels were measured using colorimetry method. The experiments were conducted in 3 replicates independently. The data were expressed as mean ± standard deviation. The independent *t* test was employed for comparisons between two groups. ∗*p* < 0.05.

Subsequently, the expression of HIF1*α* in CC cells in hypoxic and normoxic conditions was compared. The mRNA and protein levels of HIF1*α* were increased after hypoxia treatment (Figure 5(a)-5(b), all *p* <0.05). ChIP-PCR showed decreased H2K9me3 level in HIF1*α* promoter after hypoxia treatment (Figure 5(c), *p*< 0.05). shKDM4A and pcDNA3.1-HIF1*α* were introduced into CC cells simultaneously, followed by hypoxia treatment. The transfection efficiency of pcDNA3.1-HIF1*α* was determined by RT-qPCR and Western blot (Figure 5(d)-5(e)). Compared with the Hypo-2 h + shKDM4A + pcDNA3.1 group, the Hypo-2 h + shKDM4A + pcDNA3.1- HIF1*α* group had enhanced cell viability (Figure 5(f), *p*< 0.05), declined intracellular iron concentration (Figure 5(g), *p*< 0.05) and MDA level (Figure 5(h), *p*< 0.05), and elevated GSH level (Figure 5(i), *p*< 0.05). These results illuminated that HIF1*α* overexpression attenuated ferroptosis induced by KDM4A knockdown, suggesting that hypoxia upregulated KDM4A and HIF1*α* to induce CC cell resistance to ferroptosis.

### 3.6. Hypoxia Activated the HIF1/HRE Pathway and Upregulated Levels of Iron Transporters in CC Cells

**Figure 6:**
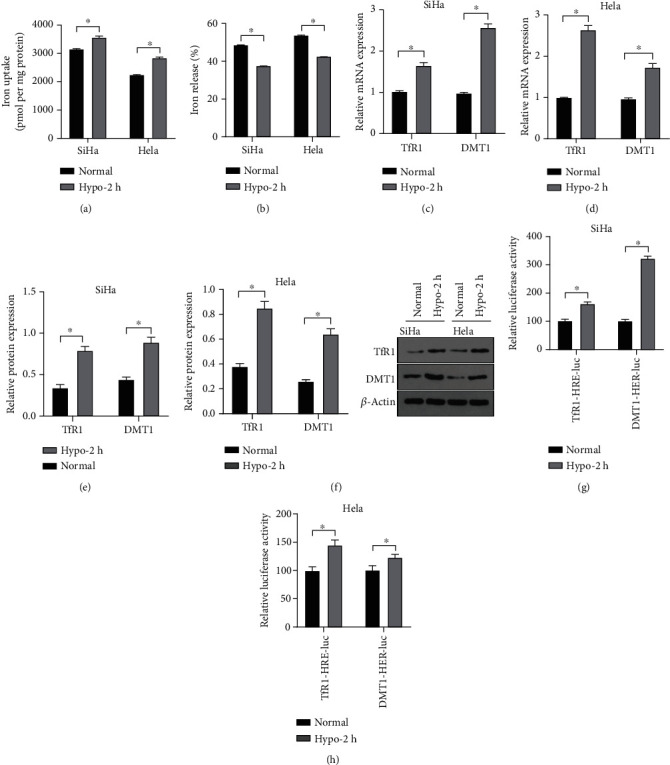
Hypoxia activated the HIF1/HRE pathway and upregulated expression of iron transporter in CC cells. SiHa and Hela cell lines were treated with hypoxia for 2 h. (A-B) Cell iron uptake and release were determined using isotope ^55^Fe tracing method; (C-D) The mRNA levels of TfR1 and DMT1 in CC cells were detected by RT-qPCR; (E-F) The protein levels of TfR1 and DMT1 in CC cells were detected by Western blot; (G) The CC cells were transfected with different luciferase plasmids to detect luciferase activity. The experiments were conducted in 3 replicates independently. The data were expressed as mean ± standard deviation. The non-paired *t* test was utilized for comparisons among groups. ∗*p* < 0.05.

As aforementioned, hypoxia treatment increased intracellular iron concentration. To explore the mechanism of HIF1*α* in iron metabolism in CC cells, we observed the alterations of iron uptake and release in CC cells using the isotope ^55^Fe tracer method. Compared with the normoxia group, the hypoxia group had significantly enhanced ability of iron uptake and weakened ability of iron release (Figure 6(a), 6(b), *p* <0.05). RT-qPCR and Western blot showed that elevated mRNA and protein levels of TfR1 and DMT1 in the Hypo-2 h group compared with those in the normal group (Figure 6(c)-6(f), all *p* <0.05). To determine whether hypoxia conditions affected the interactions among HIF1*α* and DMT1 and TfR1, luciferase activity was detected to evaluate their HRE functions. The different HRE fragments of DMT1 and TfR1 were cloned into luciferase reporter vectors and co-transfected into CC cells with pcDNA3.1-HIF1*α* plasmid. Following 2-h hypoxia, the luciferase activities of DMT1-HRE-Luc and TfR1-HRE-Luc were significantly stimulated (Figure 6(g)-6(h), *p* < 0.05).

## 4. Discussion

CC is a growing health problem around the world [33]. Ferroptosis is a newly-discovered type of programmed cell death, and CC treatment can be achieved by targeting cancer cell ferroptosis [34]. Yet hypoxia is adverse to cancer therapy [35]. The present study demonstrated that hypoxia upregulated KDM4A and downregulated H3K9me3 in the HIF1*α* promoter region to enhance HIF1*α* transcription activity and activate HRE sequence in TfR1 and DMT1 promoters, thus inducing ferroptosis resistance in CC cells.

Uncontrolled multiplication of solid tumors leads to an evident hypoxic condition status [36]. Existing research supports the idea of hypoxia being a negative influencing factor in CC treatment and reveals that hypoxia induces resistance to cisplatin in CC cells [37]. In addition to this, hypoxia also induces resistance to apoptosis [38]. Conversely, the combination of silencing GSK-3*β* and CDK1 counteracts the apoptosis resistance induced by hypoxia [39]. By performing the TUNEL assay to assess CC cell apoptosis after hypoxia treatment, we pointed out that hypoxia could induce apoptosis resistance in CC cells (Supplementary Figure 1). Ferroptosis is recently found as a form of programmed cell death characterized by iron-dependent lipid peroxide accumulation [40]. As reported earlier, hypoxia can induce resistance to ferroptosis in pancreatic cancer cells [41]. Ferroptosis of liver cancer cells can be impeded by hypoxia [42]. Our results unraveled that hypoxia for 0-2 h had no predominant impact on CC cell viability, Fe^2+^, MDA, and GSH levels. On the other hand, CC cells pre-treated with hypoxia for 2 h had decreased ferroptosis level in comparison to those pre-treated with 2-h normoxia after Erastin treatment, indicative of enhanced ferroptosis resistance in SiHa and Hela cells by 2-h pre-treatment with hypoxia.

Increased expression of KDM4A has been documented in various types of cancers [26, 43], and KDM4A serves as an oncogene in CC [32]. Our result showed the KDM4A expression was gradually increased in squamous cell carcinoma of the cervix and in SiHa and Hela cells treated with hypoxia for 0.5-12 h with the prolongation of hypoxia time. More than that, KDM4A protein levels were increased in RKO cells exposed to hypoxia [32]. And our results elicited that hypoxia upregulated KDM4A in CC cells. KDM4A depletion was reported to promote cell ferroptosis in osteosarcoma [44]. Therefore, we knocked KDM4A down in CC cells and then observed that KDM4A knockdown inhibited hypoxia-induced ferroptosis resistance in CC cells. Overall, KDM4A facilitated resistance to ferroptosis in CC cells.

H3K9me3 represents a posttranscriptional modification in regulating various biological processes [45]. There is ample evidence that KDM4A regulates HIF1*α* expressions via H3K9me3 [32]. Our result showed that HIF1*α* was highly expressed in squamous cell carcinoma of cervix exposed to hypoxia, which, not coincidentally, complied with a previous study that HIF1*α* expression was increased in CC cells under hypoxia [46]. Moreover, our result showed a positive correlation between KDM4A mRNA expression and HIF1*α* mRNA expression. KDM4A and HIF1*α* were downregulated whereas H3K9me3 was upregulated in SiHa and Hela cells after transfection with shKDM4A. KDM4A inactivation stimulates the H3K9me3 level [47]. Besides, H3K9me3 was accumulated at the HIF1*α* promoter after silencing KDM4A. KDM4A demethylates H3K9me3 and H3K9me3 accumulation leads to decreased levels of HIF1*α* [48, 49]. Collectively, KDM4A was upregulated in CC cells exposed to hypoxia and enhanced HIF1*α* transcriptional activity by downregulating H3K9me3.

HIF1*α* accumulation is implicated in the development of malignant tumors [50]. Our result showed that HIF1*α* levels were increased while H3K9me3 level in the HIF1*α* promoter region was decreased in CC cells under hypoxic conditions. Increased expression of HIF1*α* can enhance the hypoxic viability of glioma cells [51]. Accordingly, cell viability was increased, whereas iron concentration and MDA content were decreased and GSH level was increased in CC cells transfected with shKDM4A and pcDNA3.1-HIF1*α* under hypoxic conditions, suggesting that HIF1*α* overexpression counteracted the effect of KDM4A knockdown on suppressing ferroptosis resistance in CC cells. Our results elicited that hypoxia upregulated HIF1*α* expression and induced ferroptosis resistance in CC cells by upregulating KDM4A. Moreover, HIF1*α* binds to HRE, an important regulatory sequence mediating cell hypoxic response to activate downstream gene expressions [17]. HIF-1/HRE axis plays a principal role in hypoxia-induced iron uptake [29]. Our results showed that iron uptake and release capacities were increased in CC cells under hypoxic conditions. TfR1 and DMT1 participate in iron transportation and act as target genes of HIF1 [18]. Our result showed that TfR1 and DMT1 were increased in CC cells under hypoxic conditions. After transfection with pcDNA3.1-HIF1*α*, the luciferase activities of DMT1-HRE-Luc and TfR1-HRE-Luc were elevated after 2-h hypoxia treatment. Increasing evidence documented that hypoxia promotes iron uptake by regulating TfR1 and DMT1 expressions [30, 52]. Altogether, our results clarified that hypoxia upregulated the expressions of iron transporter proteins by activating the HIF-1/HRE pathway.

In conclusion, hypoxia-upregulated KMD4A stimulated HIF1*α* transcriptional activity and activated HRE sequence in TfR1 and DMT1 promoters, thus inducing resistance to ferroptosis in CC cells. KDM4A is perceived as a carcinogenic protein in CC, yet the specific functional mechanism remains elusive. Hypoxia is a basic and widespread stimulator engaged in various physiological and pathological processes and stimulation of cells, tissues, organs, and physiological systems in the human body [53, 54]. There is no doubt that hypoxia and cancer occurrence and development are closely linked to each other [55]. Hypoxia provokes complex cellular changes, in which HIF-1 activation plays a dominant role [56]. Modern studies concerning hypoxia see HIF-1 as a critical nuclear transcription factor with regulatory properties in expressions of hypoxia target genes and intracellular oxygen environment [57, 58]. By purifying HIF1 protein from Hela cells, Semenza et al. delineated that HIF1 is a dimer structure consisting of HIF1*α* and HIF1*β* subunits, and the former is regulated by oxygen [59, 60]. Hundreds of target genes of HIF1 have been identified in every aspect of tumorigenesis and development, such as angiogenesis, lymphangiogenesis, cell invasion and metastasis, and tumor metabolism [61]. In recent years, HIF1 has also been found in the regulation of tumor stem cells [62]. Our results indicated a regulatory relationship between KDM4A and HIF-1. Further exploration into their interactions could reveal the regulatory mechanism of KDM4A in tumor cells.

Ferroptosis is a pattern of cell death discovered recently in close relation to the imbalance of cellular redox homeostasis [63, 64]. An existing study has manifested that tumor cells need higher iron levels than normal cells to facilitate growth, which makes cancer cells more susceptible to ferroptosis [65]. Scholars have put forward the strategy of treating tumors with ferroptosis since its discovery. This study, with ferroptosis resistance as the breakthrough point, unveiled that HIF1*α* affected ferroptosis resistance in CC cells. This study also implied that hypoxia induced alterations in iron metabolism in CC cells, including elevation in iron uptake and release, yet the mechanism of changes regarding iron release requires further investigation. HIF plays a transcriptional and regulatory role in multiple aspects of tumorigenesis. However, except HIF-1, the engagement of HIF-2 in the regulatory mechanism of hypoxia in CC remains to be addressed in future studies.

## Figures and Tables

**Table 1:** Primer sequences.

Gene	Forward 5'-3'	Reverse 5'-3'
*HIF1α*	GAACGTCGAAAAGAAAAGTCTCG	CCTTATCAAGATGCGAACTCACA
*KDM4A*	ATCCCAGTGCTAGGATAATGACC	ACTCTTTTGGAGGAACAACCTTG
*TfR1*	ACCATTGTCATATACCCGGTTCA	CAATAGCCCAAGTAGCCAATCAT
*DMT1*	TGGAGATCATGGGGAGTCTG	AAGAAAACCTGGTCCGGTGAA
*β-Actin*	CATGTACGTTGCTATCCAGGC	CTCCTTAATGTCACGCACGAT

Note: HIF1α, hypoxia-inducible factor 1*α*; KDM4A, lysine (K)-specific demethylase 4A; TfR1, transferrin receptor 1; DMT1, divalent metal transporter 1.

## Data Availability

The data that support the findings of this study are available from the corresponding author upon reasonable request.

## Supplementary Materials

Supplementary Materials Supplemtal Figure 1. TUNEL staining was implemented to detect the function of hypoxia and normoxia on cell apoptosis resistance.
