# Supplementary material for: Hypoxia Enhances HIF1α Transcription Activity by Upregulating KDM4A and Mediating H3K9me3, Thus Inducing Ferroptosis Resistance in Cervical Cancer Cells
Source: Stem Cells Int. 2022 Mar 5;2022:1608806. doi: 10.1155/2022/1608806 (PMC8917951; doi:10.1155/2022/1608806)
Supplement: Supplementary Materials — Supplemtal Figure 1. TUNEL staining was implemented to detect the function of hypoxia and normoxia on cell apoptosis resistance. [file 1608806.f1.docx]

**Hypoxia induces resistance to apoptosis in cervical cancer cells**

**Methods: Cell apoptosis detected by TUNEL staining**

Cell apoptosis was detected using the TUNEL Apoptosis Detection kit (Roche, Indianapolis, IN, USA). In brief, SiHa and Hela climbing sheets were prepared under different conditions, fixed with 4% paraformaldehyde for 30 min, washed with PBS 3 times, and incubated with 0.3% Triton 100 for 5 min at room temperature and with TUNEL detection solution for 1 h. Following 3 PBS washes, cells were added with DAPI solution and incubated for 2 min at room temperature, followed by 3 PBS washes. Cells were subsequently observed under a fluorescent microscope. Five non-overlapping visual fields were selected at random for per section to calculate the positive apoptotic cell percentage (%), which was shown as mean ± standard deviation.

**Results: Hypoxia induced resistance to apoptosis in CC cells**

The apoptosis of SiHa and Hela cells treated with 2-h normoxia or hypoxia and incubated with Erastin for 24 h was detected by TUNEL staining, which showed an increase in cell apoptosis in the Normal + Erastin group or Hypo-2h + Erastin group relative to that in the Normal group or Hypo-2h group, indicating that ferroptosis inducer Erastin facilitated cell apoptosis. In comparison with the Normal + Erastin group, the Hypo-2h + Erastin group showed decreased apoptosis rates (Supplementary figure 1, all *p* < 0.05), suggesting that hypoxia impeded cell apoptosis induced by Erastin, which meant that hypoxia induced apoptosis resistance in CC cells.


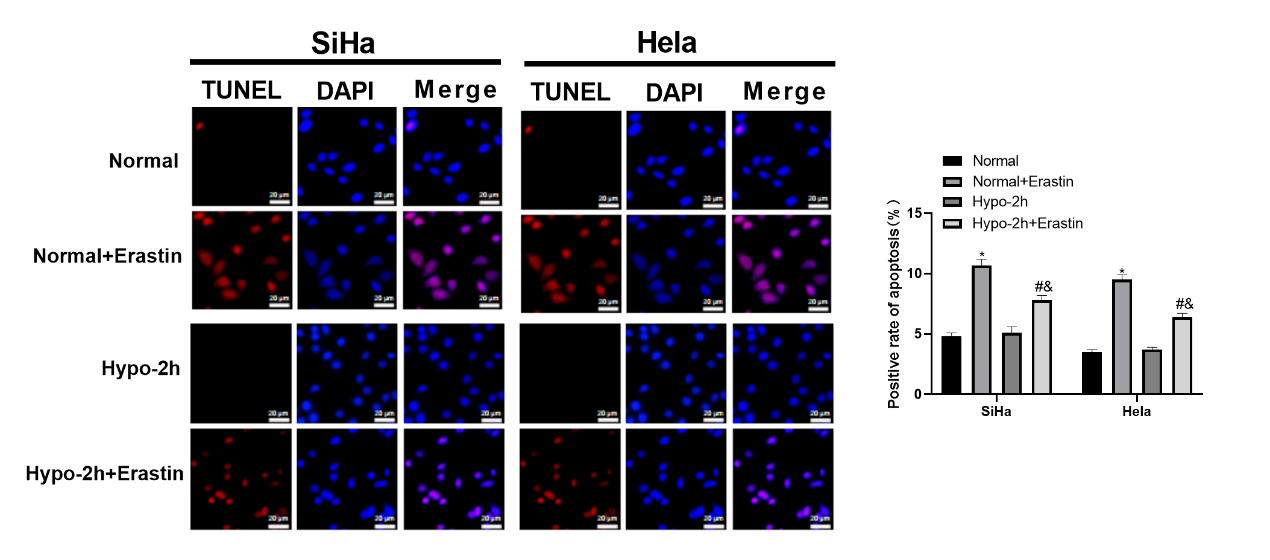


TUNEL staining was implemented to detect the function of hypoxia and normoxia on cell apoptosis resistance. Data were expressed as mean ± standard deviation. * vs. the Normal group, *p* < 0.05; & vs. the Normal + Erastin group, *p* < 0.05; # vs. the Hypo-2h group, *p* < 0.05.
